# Melatonin and arbuscular mycorrhizal fungi synergistically improve drought toleration in kiwifruit seedlings by increasing mycorrhizal colonization and nutrient uptake

**DOI:** 10.3389/fpls.2022.1073917

**Published:** 2022-12-01

**Authors:** Hui Xia, Chunguo Yang, Yan Liang, Zunzhen He, Yuqi Guo, Yuxuan Lang, Jie Wei, Xinbo Tian, Lijin Lin, Honghong Deng, Jin Wang, Xiulan Lv, Dong Liang

**Affiliations:** ^1^ College of Horticulture, Sichuan Agricultural University, Chengdu, China; ^2^ College of Life Sciences, Inner Mongolia University, Hohhot, China

**Keywords:** arbuscular mycorrhizal fungi, melatonin, kiwifruit, drought tolerance, biomass, nutrient uptake

## Abstract

Kiwifruit is a vine fruit tree that is vulnerable to water deficiency due to its shallow root system and large leaves. Although mycorrhizal inoculation and melatonin application has been proved to improve plants drought tolerance, their interaction effects are still unclear. In this study, arbuscular mycorrhizal (AM) fungi incubation and melatonin (MT) irrigation were applied to kiwifruit seedlings alone or in combination to investigate their effect on drought tolerance. The results revealed that AM had more effect on promoting root biomass, water use efficiency, and uptake of nitrogen, phosphorus and iron. While MT was more effective in promoting shoot biomass and antioxidant enzyme activities to remove reactive oxygen species accumulation. Moreover, MT supplementary significantly increased the AM colonization, spore density and hyphal length density in roots. Therefore, combined application of AM fungi and MT had additive effects on improvement biomass accumulation, increasing chlorophyll content, photosynthetic efficiency, catalase activity, and decreasing malondialdehyde accumulation under drought stress, thus promoting plant growth and alleviating the drought damage to plant. These results provide guidance for AM and MT combined application to improve abiotic resistance in plants.

## Introduction

Water scarcity caused by global warming is becoming more common, posing a huge challenge to agricultural production, and resulting in large-scale crop losses and food shortages for mankind (Zhang et al., 2018; Rivero et al., 2022). Plants have evolved a series of mechanisms to cope with drought stress, such as prolonged root growth, regulating stomata movement, and maintaining the osmotic balance of cells (Mathur and Roy, 2021). In addition, plants also use a variety of enzymatic and non-enzymatic antioxidants to efficiently and rapidly remove reactive oxygen species (ROS) (Fang and Xiong, 2015; Khan et al., 2021).

Plant growth-promoting rhizobacteria, such as arbuscular mycorrhizal (AM) fungi, play an important role in alleviating drought stress in plants (Vurukonda et al., 2016; Porter et al., 2020; Abdelaal et al., 2021). AM, act as an extension of the plant root system, provide their host plants with access to soil water and nutrients through far-reaching extra radical hyphae, while fungi are provided with carbon from host plants (Gutjahr and Paszkowski, 2013; Frew et al., 2022). Most importantly, this symbiosis relationship creates a functionally active ecosystem that influence nutrient cycling, decomposition, soil aggregation, belowground biodiversity, and plant community ecology (Kaushal, 2019; Zhang et al., 2020). Under drought stress, AM reduced the damage of free radicals by promoting nutrient absorption capacity, improving root function, maintaining leaf photosynthetic efficiency (Lenoir et al., 2016). In addition, extrapular-root mycelium formed a huge mycelium network in the soil, increasing root xylem conductance and improving the adaptability of water transport (Abdelaal et al., 2021; Sun et al., 2022). Meanwhile, the polysaccharides produced by fungi also have the ability to increase mineral strength and retain water (Hooker et al., 2007).

Melatonin (N-acetyl-5-methoxy-tryptamine), as a master regulator, participant in plant development and protects plant against almost all abiotic stresses, such as drought, salt damage, heavy metals, UV radiation, high temperature, cold damage, etc. (Arnao and Hernández-Ruiz, 2019; Sun et al., 2021). Melatonin ameliorates the adverse effects of drought by regulating morphological, physiological and redox regulatory processes, and a variety of regulatory mechanisms have been explored in recent years (Tiwari et al., 2021). Most fundamentally, melatonin has the strong antioxidant activity to remove ROS and free radicals caused by stress, by improving antioxidant enzymes activities, such as ascorbic acid, ascorbate peroxidase, superoxide dismutase (SOD), catalase (CAT) and peroxidase (POD), and the content of antioxidants such as flavonoids (Huang et al., 2019). Melatonin also affects the nutrients absorption from soil by regulating the activity and expression of nutrient transporters (Xia et al., 2021; Arnao et al., 2022; Du et al., 2022; Sun et al., 2022). Melatonin was also found can solve the replant problem by altering the composition of bacterial and fungal communities (Li et al., 2018), and promoted the primary root growth in *Arabidopsis* (Yang et al., 2021).

Kiwifruit (*Actinidia chinensis* Planch.) is an emerging fruit tree grown worldwide, but it is more vulnerable to drought than other fruit trees because of its shallow, fleshy root system and large, hairy leaves (Liang et al., 2019). It has been confirmed that melatonin pretreatment can increase the biomass of kiwifruit seedlings by improving leaf photosynthetic capacity and carbon fixation, and improve the drought tolerance of kiwifruit seedlings by promoting root elongation and growth and increasing antioxidant content (Liang et al., 2019; Xia et al., 2020). However, whether melatonin and AM have synergistic effects on drought resistance in plants is still unknown. In this study, AM fungi and melatonin were applied to kiwifruit seedlings alone or in combination to investigate the effect on plant growth, photosynthesis, antioxidant enzyme activity, nutrient absorption, in order to further find a better scheme to improve the drought tolerance of kiwifruit for field cultivation.

## Materials and methods

### Plant materials preparation and treatment

Kiwifruit seeds (*Actinidia chinensis*) were planted in a tray containing mixed matrix and grown in a light incubator after germination treatment according to the method of Liang et al. (2019). When grow to 6-true leaves, the seedlings were then transformed into pots (18*20*20 cm) containing 1.5 kg mixed soil (garden soil: peat soil: perlite = 2:1:1, V/V/V), and placed in a greenhouse at Chengdu Campus of Sichuan Agricultural University (103°51′ E, 30°42′ N) under natural light and temperature conditions. Each pot planted 2 seedlings. After two weeks of cultivation for adaption, 96 pots of uniform seedlings were selected and evenly divided into two groups for normal watering treatment and drought treatment, and each contained four treatments, including 1) the control (CK), treated with water; 2) inoculated AM fungi in soil (AM); 3) root irrigation with melatonin solution (MT); and 4) combination treatment with melatonin and AM (AM+MT) under drought or normal watering condition. First, 10.0 g sterilized or non-sterilized mycorrhizal (*Rhizophagus intraradices*), purchased from Nanjing Cuijingyuan Biotechnology Co., Ltd. was inoculated in the soil for 4 weeks to form mycorrhizas. Then seedings were irrigated with 200 mL water or 100 μM melatonin solution for twice at three-day intervals at nightfall, followed by water control treatment. The melatonin concentration was chosen based on our previous study (Xia et al., 2020). Soil moisture content was maintained at 50~55% of maximum water holding capacity for drought treatment using soil weighing method, while 80~85% of maximum water holding capacity for watering treatment. Each treatment included 12 pots (24 seedlings), seedlings in two pots were collected as a repeat and, repeated 6 times. Samples were collected at 12 d of drought treatment. The mature leaves (from 3rd to 5th) were sampled and frozen with liquid nitrogen and stored at -80°C for physiological index determination. Then the whole plants were removed from the soil and cleaned for biomass determination.

### The arbuscular mycorrhizal fungi tested

The harvested fresh seedling roots were cleaned and stained with 0.05% trypan blue, followed by washing and decolorization with lactic acid glycerol solution (lactic acid, glycerol, distilled water = 1:1:1 (v/v/v), and then the roots were observed under a microscope (50 per plant, 5 per treatment). AM colonization was calculated according to the method of Biermann and Linderman (1981). The grid line intersection method of Yang et al. (2020) was used to determine spore density and hyphal length density.

### Plant growth parameters determination

After being removed from the soil and cleaned, the plant height, root length, and stem diameter were determined with a meter ruler or vernier caliper. The dry weight of the shoot and root were weighed after drying in an oven at 105°C, then baked at 80°C until constant weight. all data contained three biological repeats.

### Pigment content determination

The determination of chlorophyll content was improved according to the method of Liang et al. (2019). The 0.1 g fresh leaves were cut into pieces and put into a centrifuge tube containing 8 mL 80% acetone, and kept in dark for 48 h, shacked 3-4 times. The absorbance values of the supernatant at 663, 645 and 470 nm were determined by UV/V spectrophotometer (UV-2550). The content of total chlorophyll, chlorophyll a, and chlorophyll b was calculated.

### Determination of gas exchange parameters

A portable gas exchange system (LI-6400, Li-Cor Inc., USA) was used to determine photosynthetic gas exchange parameters between 9:00 am and 11:00 am, including net photosynthetic rate (Pn), transpiration rate (Tr), intercellular CO_2_ concentration (Ci), stomatal conductance (Gs) and water use efficiency (WUE). A red/blue LED light source, a constant flow rate of 500 mL·min^-1^ and CO^2^ concentration of ca. 400 μmol·mol^-1^ under a PAR of 1000 μmol·m^-2^s^-1^ were used. At least 3 seedlings were measured in each treatment.

### Determination of physiological indexes

The relative electrical conductivity (REL) and the content of malondialdehyde (MDA), proline, soluble protein and soluble sugar were determined according to the methods of Liu et al. (2020). Regards to antioxidant enzyme activity, the frozen leaf samples were homogenized in liquid nitrogen and extracted with an extraction buffer composed of 100 mM potassium phosphate buffer (pH 7.8), 0.1 mM EDTA and 10 mM ascorbic acid. The supernatant after centrifugation was used to determine the activities of superoxide dismutase (SOD), catalase (CAT) and peroxidase (POD) (Cakmak and Marschner, 1992).

### Determination of nutrients content

0.2 g of dry leaves were digested with 10 mL nitric acid and 2 mL perchloric acid, and boiled until white crystal appeared in graphite digestion machine (Xiyang Instrument Co., Ltd., Shanghai, China) (about 2 h). After cooling, the volume was fixed to 25 mL with ultrapure water for the determination of the content of each element. The content of nitrogen (N), phosphorus (P) and potassium (K) was determined by Kjeldahl nitrogen analyzer (KDN-04C Tuopuyunnong Technology Co., Ltd., Zhejiang, China), molybdenum-antimony resistance colorimetry, and flame photometer (INESA Scientific Instrument Co., Ltd, Shanghai, China), respectively. The content of iron (Fe), manganese (Mn), copper (Cu) and zinc (Zn) were determined by atomic absorption spectrophotometry using inductively coupled plasma emission spectrometry (ICP-OES, Optima 8000, PerkinElmer, USA).

### Gene expression assay

Total RNA was extracted using the Mini RNA Isolation I Kit (Beijing Tianmo Sci & Tech Development Co., Ltd, China) according to the manufacturer’s instructions. The first strand cDNA was obtained by reverse transcribing RNA with PrimeScript™ RT reagent Kit with gDNA eraser (Perfect Real Time) (Takara, Japan). The gene-specific primers were designed using Primer Premier5 and synthesized by Tsingke Biotechnology Co., Ltd (Beijing, China). *Actin* was used as the reference (Xia et al., 2020). Quantitative Realtime PCR was performed on the CFX96 Real-Time System C1000 Thermal Cycler (Bio-RAD, Hercules, CA, USA) using an SYBR^®^ Premix Ex Taq™ II (Tli RnaseH Plus) kit (TaKaRa, Japan). The reaction conditions were as follows: 95 °C for 30 s, followed by 40 cycles of 95 °C for 5 s, and at 52 °C to 55 °C for 30 s. Each sample was subjected to three replicates. The 2^- ΔΔCT^ method was used to calculate the relative mRNA expression level.

### Statistical analysis

Data were expressed as mean ± standard deviation (SD). One-way analysis of variance (ANOVA) and Tukey’s *Post Hoc* test (*p* < 0.05) was used to test the difference between the treatments using SPSS.

## Results

### AM fungal growth and mycorrhizal colonization

**Figure 1:**
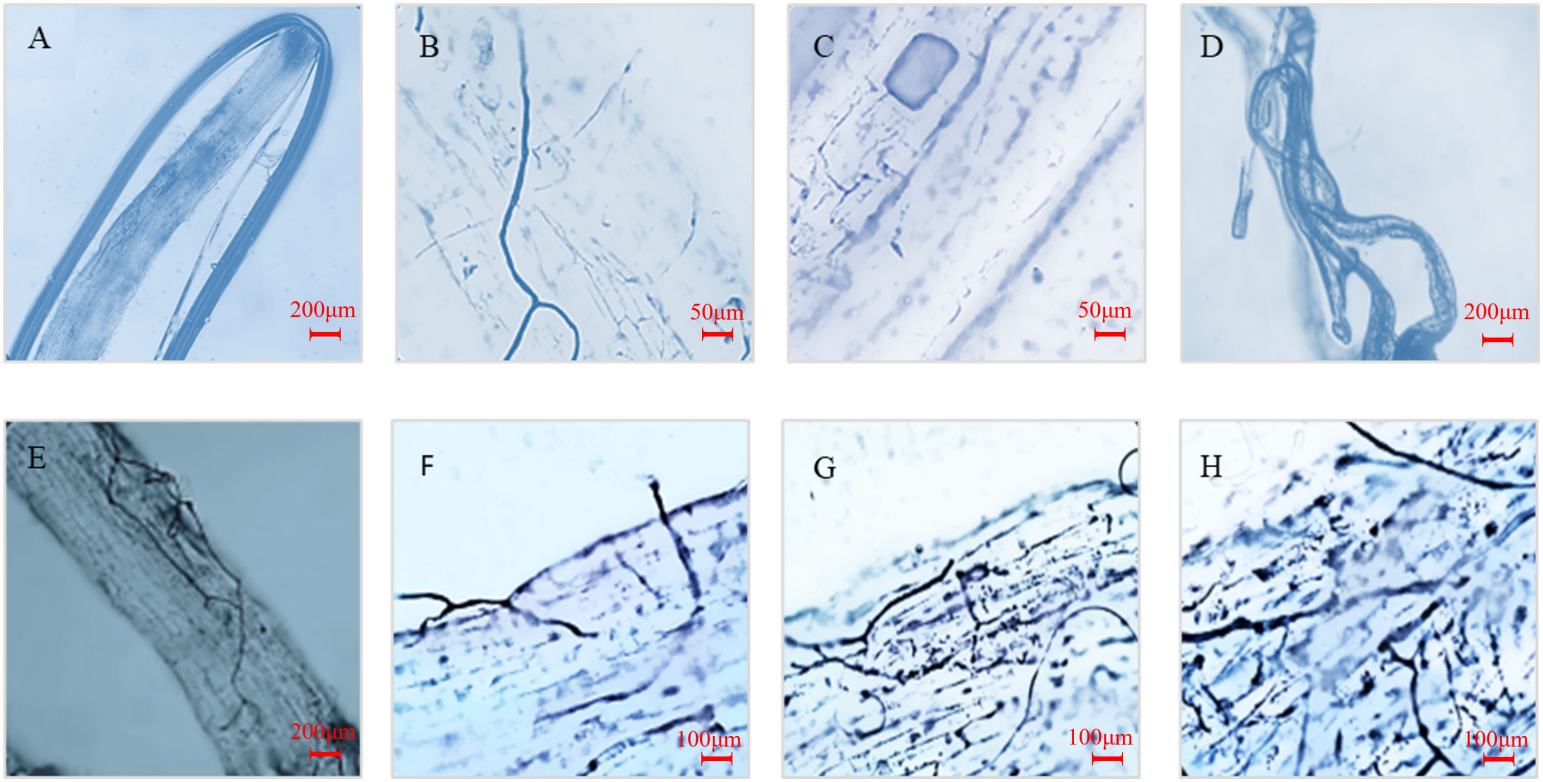
Mycorrhizal phenotype of seedling roots inoculated with *R. intraradices* after 12 d of drought treatment. **(A)** Roots of seedlings not inoculated with AM fungi; **(B–D)** Hyphae, vesicles and arbuscules in roots after mycorrhizal infection; **(E, F)**: Mycorrhizal infection of roots without external melatonin treatment; **(G, H)** Mycorrhizal infection of roots treated with external melatonin.

The infection of AM fungi with or without MT on the root system of kiwifruit seedlings was observed by trypan blue staining (**Figure 1**) The results showed that the roots of uninoculated seedlings were not infected by AM fungi, while the roots of seedlings inoculated with AM fungi for 50 d showed plenty of mycelia, vesicles and typical arbuscule structures after inoculation with AM fungi. This indicated that AM fungi and kiwifruit seedling roots established a good symbiotic relationship.

Encouragingly, melatonin treatment effectively increased root infection by inoculating AM fungi. The infection rate increased by 14.6% under normal watering condition and 37.4% under drought condition. In addition, the spore density and hyphal length density increased by 40.6% and 40.7% under drought, respectively (**Table 1**). The results suggest that melatonin played a role to promote the colonization of *R. intraradices* in the root, thus forming a symbiotic relationship.

**Table 1:** Effects melatonin application on AM colonization, spore density and hyphal length density in seedling roots inoculated with *R. intraradices*.

Treatments		AM fungal status		
		MC (%)	SPD (number/g)	HLD (m/g)
WW	-MT	65.7 ± 0.76b	6.27 ± 0.27c	2.34 ± 0.08b
	+MT	75.3 ± 2.33a	7.58 ± 0.09b	3.77 ± 0.17a
DR	-MT	52.9 ± 1.66c	6.50 ± 0.13c	1.62 ± 0.03c
	+MT	72.7 ± 2.99a	9.14 ± 0.16a	2.28 ± 0.04b

The values (means ± SD, n = 6) followed by different letters in the same column reprensent significant difference at p < 0.05 assayed by Tukey’s Post Hoc test. MC, AM colonization; SPD, spore density; HLD, hyphal length density; WW, well-watering; DR, drought stress; -MT, non-melatonin application; +MT, melatonin application.

### Effect of treatments on plant growth and biomass

**Figure 2:**
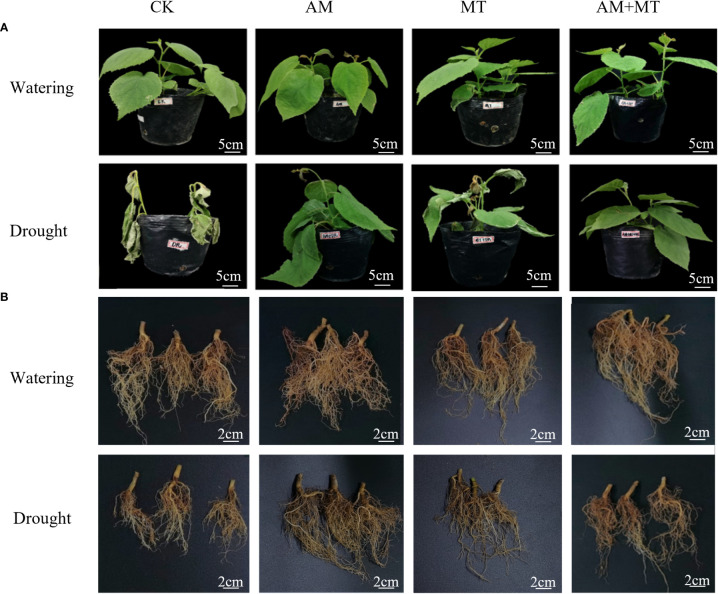
Effect of treatment on plant growth of above-ground **(A)** and under-ground **(B)** parts under well watering and drought condition. Treatments included CK (the control), AM (inoculated with arbuscular mycorrhiza), MT (irrigated with 100 μM melatonin), and AM+MT (treated with melatonin and arbuscular mycorrhiza).

After 12 d of drought treatment, the seedling leaves were seriously dehydrated and wilted, and roots, especially lateral roots, were significantly reduced and shortened (**Figures 2A, B**). AM, MT and AM+MT treatments apparently alleviated the morphological damage caused by drought, exhibited reduced leaf wilting and increased root length and abundance compared with CK.

**Figure 3:**
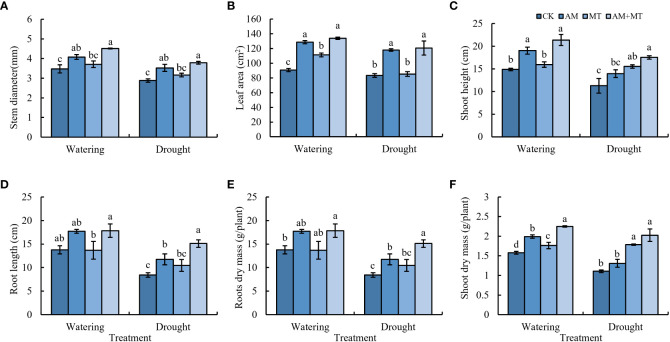
Effect of treatments on plant growth, including stem diameter **(A)**, leaf area **(B)**, shoot length **(C)**, root length **(D)**, root dry mass **(E)**, shoot dry mass **(F)**. Data are presented as meant **±** SD (*n* = 6), different letters indicate significant differences between treatments at *p* < 0.05 by Tukey’s *Post Hoc* test. Treatments included CK (the control), AM (inoculated with arbuscular mycorrhiza), MT (irrigated with 100 μM melatonin), and AM+MT (treated with melatonin and arbuscular mycorrhiza).

Accordingly, the application of AM and MT improved seedling growth under drought stress. AM and AM+MT treatments significantly increased shoot length, stem diameter, leaf area, root length, and shoot and root dry mass under well-watering condition, while MT alone application only increased the leaf area and shoot dry mass (**Figure 3**). AM+MT treatment had best effect in increase shoot and root dry mass by 25% and 41.18%, respectively, compared with CK (**Figures 3E, F**).

### Effect of treatments on photosynthetic pigments and gas exchange parameters

**Figure 4:**
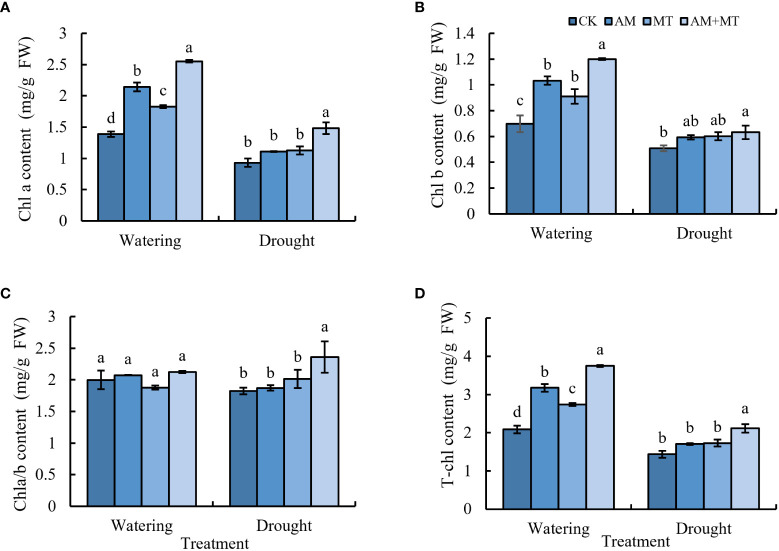
The content of chlorophyll a **(A)**, chlorophyll b **(B)**, total chlorophyll **(C)**, and chlorophyll a/b **(D)** in kiwifruit seedlings under well-watering or drought condition. Data are presented as meant **±** SD (*n* = 6), different letters indicate significant differences between treatments at *p* < 0.05 by Tukey’s *Post Hoc* test. Treatments included CK (the control), AM (inoculated with arbuscular mycorrhiza), MT (irrigated with 100 μM melatonin), and AM+MT (treated with melatonin and arbuscular mycorrhiza).

Under drought stress, the content of chlorophyll a, b and total chlorophyll decreased greatly (**Figures 4A-C**). Application of AM fungi, MT alone or combined significantly increased the content of chlorophyll a, b and total chlorophyll by 25.93%, 11.11% and 35.19%, respectively, compared with CK. Results suggested that AM+MT had the best effect on the enhancement of chlorophyll content under drought condition.

**Figure 5:**
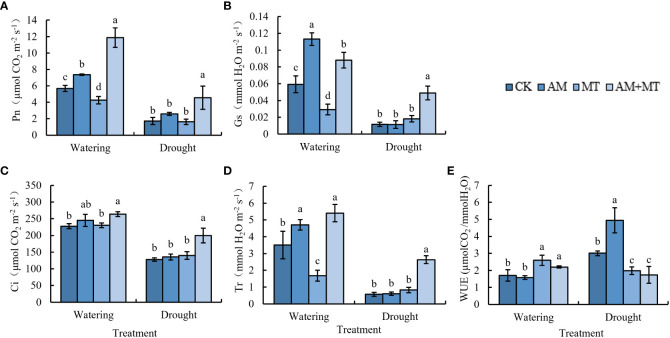
Effect of treatments on gas exchange parameters in kiwifruit seedlings under well-watering or drought stress, including net photosynthetic rate **(A)**, stomatal conductance **(B)**, intercellular CO_2_ concentration **(C)**, transpiration rate **(D)** and water use efficiency **(E)**. Data are presented as meant **±** SD (*n* = 6), different letters indicate significant differences between treatments at *p* < 0.05 (Tukey’s *Post Hoc* test). Treatments included CK (the control), AM (inoculated with arbuscular mycorrhiza), MT (irrigated with 100 μM melatonin), and AM+MT (treated with melatonin and arbuscular mycorrhiza).

Gas exchange parameters, Pn, Gs, Tr, Ci, and WUE were determined. Values of all parameters were significantly lower under drought stress than under well watering. Application of AM and MT alone didn’t have significant effect on Pn, Gs, Tr and Ci. While when combined AM and MT applied together, values of Pn, Gs, Ci and Tr were increased by 180.96%, 168.72%, 38.55% and 218.99% compared with CK, respectively (**Figure 5**). AM treatment significantly improved the WUE value compared with other treatments.

### Effect of treatments on MDA, REL and osmotic substances

**Figure 6:**
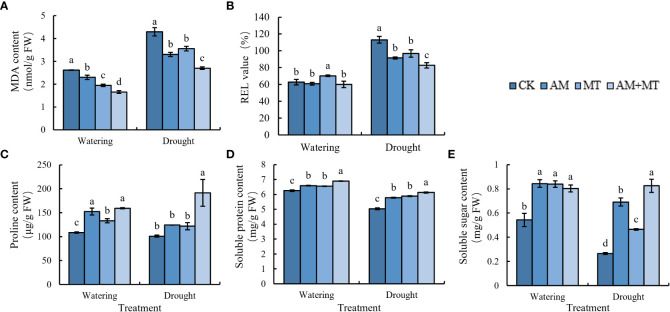
Changes of MDA **(A)**, REL **(B)**, proline **(C)**, soluble protein **(D)**, and soluble sugar **(E)** in kiwifruit seedlings under well watering and drought stress. Data are presented as meant **±** SD (*n* = 6), different letters indicate significant differences between treatments at *p* < 0.05 by Tukey’s *Post Hoc* test. Treatments included CK (the control), AM (inoculated with arbuscular mycorrhiza), MT (irrigated with 100 μM melatonin), and AM+MT (treated with melatonin and arbuscular mycorrhiza).

MDA and REL are important indexes to reflect the damage degree of plant cell membrane caused by stress. Under well-watering condition, inoculation of AM fungi, MT application and their combination decreased MDA content, while maintained the relative electronic leakage (REL) in leaves. Water deficit dramatically increased MDA content and REL in leaves, suggestion seedlings were seriously stressed. Pretreatment of AM fungus, MT and their combined significantly reduced the increase of MDA content and REL value compared with the control. In addition, combined application of AM and MT had the best alleviating effect on drought stress than alone (**Figures 6A, B**).

The contents of proline, soluble sugar and soluble protein in leaves decreased due to drought stress (**Figure 6C**). After treatment with AM, MT and AM+MT, they all increased. Soluble sugar content increased most with more 9%, especially in treatment with MT, and proline content increased most in AM+MT groups by 28.8% (**Figures 6D, E**).

### Effect of treatments on antioxidant enzyme activities and gene expression

**Figure 7:**
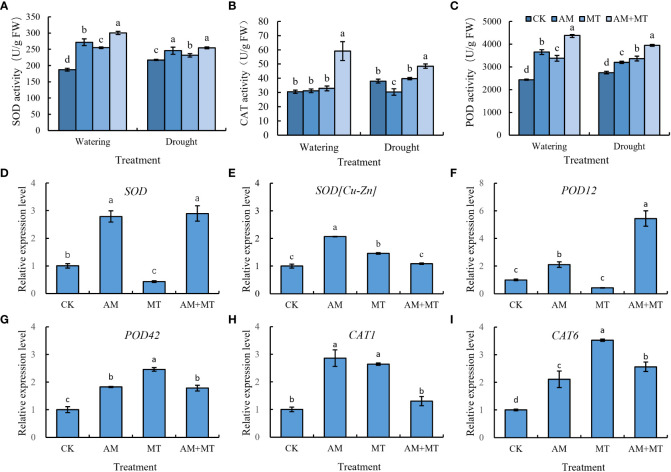
Changes of antioxidant activity of SOD **(A)**, CAT **(B)**, and POD **(C)**, and gene expression **(D–I)** in kiwifruit seedlings under well watering and drought stress. Data are presented as meant **±** SD (*n* = 3), different letters indicate significant differences between treatments at *p* < 0.05 by Tukey’s *Post Hoc* test. Treatments included CK (the control), AM (inoculated with arbuscular mycorrhiza), MT (irrigated with 100 μM melatonin), and AM+MT (treated with melatonin and arbuscular mycorrhiza).

Treatments of AM, MT and AM+MT significantly increased the activities of SOD and POD in kiwifruit seedling leaves under well-watering condition. Drought stress increased the activity of antioxidant enzymes. Under drought stress, AM, MT and AM+MT treatments increased SOD activity by 12.91%, 12.03% and 19.53%, and improved POD activity by 7.96%, 8.13% and 11.41% compared with CK. AM and MT alone had little effect on CAT activity, while AM+MT increased CAT activity by 13.18%. The results showed that the combined application of AM and MT had the strongest effect on improving the activities of SOD, POD and CAT (**Figures 7A-C**).

After 12 d of drought stress, the expression of all antioxidant enzyme genes (*SOD*, *SOD[Cu-Zn]*, *POD12*, *POD42*, *CAT1* and *CAT6*) in mycorrhizal plants was higher than that in non-mycorrhizal plants. AM+MT treatments significantly affected the expression of *SOD* and *POD12* in leaves, while had no significant effect on the expression of *Cu/Zn-SOD* and *CAT1* in leaves (**Figures 7D-I**).

### Effect of treatments on nutrient absorption

**Figure 8:**
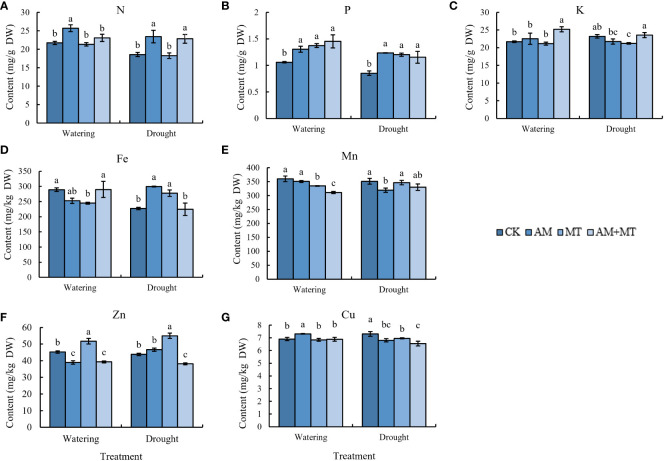
Content of N **(A)**, P **(B)**, K **(C)**, Fe **(D)**, Mn **(E)**, Zn **(F)**, and Cu **(G)** in leaves of kiwifruit seedling under well-watering or drought stress. Data are presented as meant **±** SD (*n* = 6), different letters indicate significant differences between treatments at *p* < 0.05 by Tukey’s *Post Hoc* test. Treatments included CK (the control), AM (inoculated with arbuscular mycorrhiza), MT (irrigated with 100 μM melatonin), and AM+MT (treated with melatonin and arbuscular mycorrhiza).

Under watering condition, AM and AM+MT treatments significantly increased the content of N and P in kiwifruit seedling leaves, while the content of K was not significantly changed (**Figures 8A-C**). Under drought stress, the content of N and P in CK decreased significantly, AM and AM+MT treatments significantly increased the contents of N and P. The effect of AM on N content is greater than that of MT, and MT on P content is greater than that of AM.

For mineral nutrients, AM increased the content of Cu, but decreased the absorption of Mn and Zn, while MT increased the content of Zn under well-watering condition. Under drought stress, AM and MT treatment alone increased the content of Fe and Zn, but decreased Cu content (**Figures 8D-G**).

### Effect of treatments on expression of PHT1 genes

**Figure 9:**
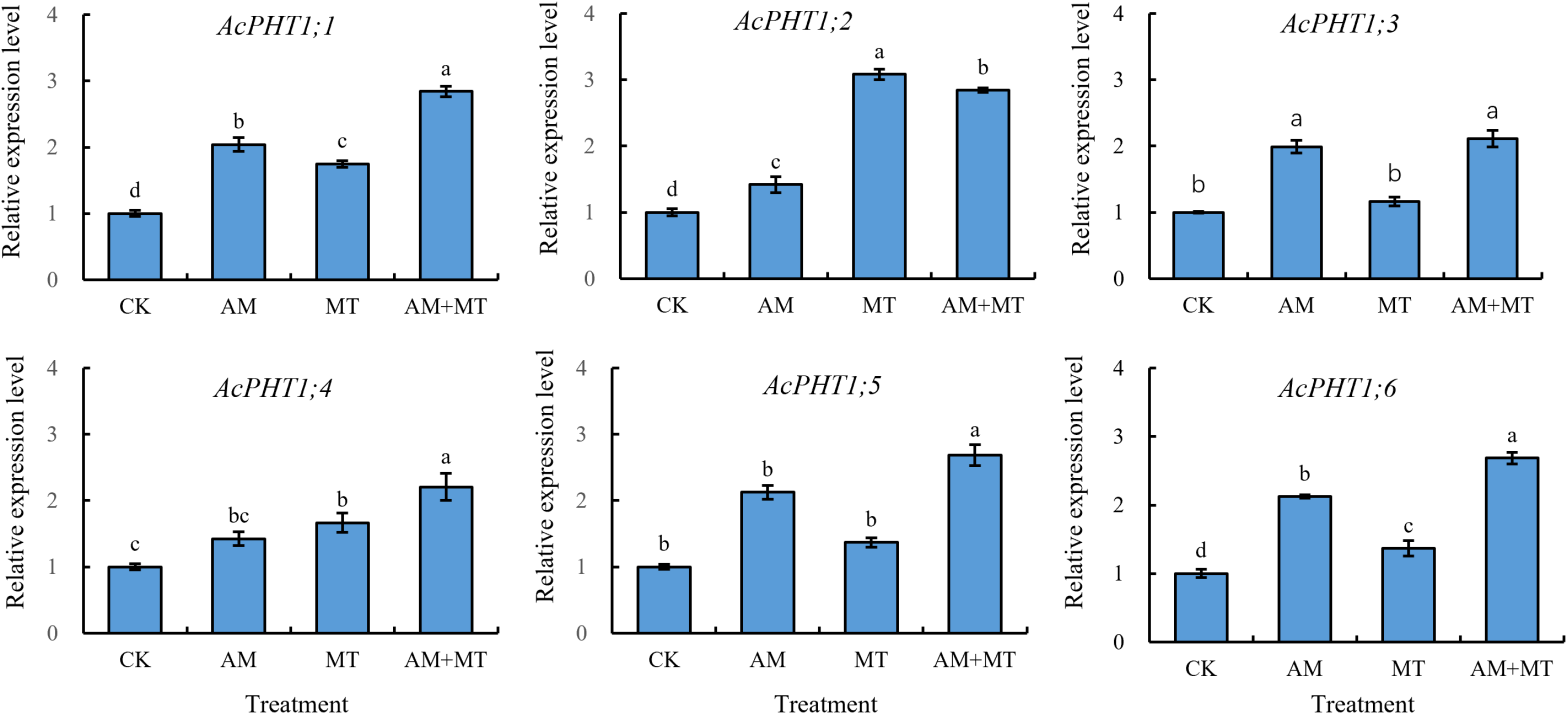
The relative expression profiles of phosphorus transport genes in the kiwifruit seedling under drought stress. Data are presented as meant **±** SD (*n* = 6), different letters indicate significant differences between treatments at *p* < 0.05 by Tukey’s *Post Hoc* test. Treatments included CK (the control), AM (inoculated with arbuscular mycorrhiza), MT (irrigated with 100 μM melatonin), and AM+MT (treated with melatonin and arbuscular mycorrhiza).

Phosphorous transporter class 1 (PHT1) serves as the primary transporters involved in phosphorous uptake from the rhizosphere (Sun et al., 2012). A total of 11 PHT1 genes were identified in *Actindia* using transcriptome data previously obtained by our team. Six PHT1s were detected expression during drought treatment. MT and AMF inoculation significantly induced the expression of the predicted six PHT1s under drought conditions. Both AMF and MT treatments up-regulated the expression of *AcPHT1;1*, *AcPHT1;2, and AcPHT1;6*. Furthermore, the combined treatment of MT and AMF significantly up-regulated the expression of all six genes, and the effect was significant than that of treatment alone (**Figures 9A-F**).

## Discussion

Improving crop drought resistance and reducing yield loss have always been the focus of global agricultural production (Zhang et al., 2021). In addition to breeding resistant varieties and mining resistance genes afford by scientists, improving plant water use efficiency and resistance by use of exogenous stimulants or bacterial agents seems to be more effective way to conquer abiotic stresses brought about by climate change and thus is favored by farmers (Farooq et al., 2019).

Increasing evidence studies have demonstrated that MT application and AM fungi inoculation have positive effects on plant growth under drought conditions (Liu et al., 2020; Bolin et al., 2022). AM fungi alleviate drought stress through several mechanisms (Abdel-Salam et al., 2017). One most important is that AM fungi induce changes in plant root structure, especially the increase of root length, root surface area, root volume and root tip number. Improvements in the root structure of mycorrhizal plants allows extraparal hyphae to extend beyond the barren areas of the plant rhizosphere, thus more efficiently absorbing water and low-mobility mineral nutrients under water scarcity conditions (Müller, 2021; Persi and Maherali, 2021). Melatonin, on the other hand, promotes the growth of lateral root (Bian et al., 2021). In this study, both melatonin and AM application alone reduced the decline of dry biomass (**Figure 2**). However, inoculation with AM fungi had a greater effect on increasing root biomass than melatonin, melatonin increased shoot biomass significantly, and their combination had the strongest effect, which was consistent with previous study (Liu et al., 2020). These results indicated that AM fungi and melatonin may drive different mechanisms to alleviate drought stress (Yang et al., 2020).

Plants rely on chlorophyll for photosynthesis to accumulate biomass (Nozue et al., 2016). Under drought stress, their cells undergo plasmid lysis, chloroplast membrane rupture, ultrastructural damage, and chlorophyll synthesis decreases and degradation accelerates, leading to the reduction of its content and photosynthetic capacity, which further affects plant growth and yield (Tambussi et al., 2000; Munné-Bosch et al., 2001). In this study, melatonin treatment and AM fungi inoculation increased the content of chlorophyll a, and b in kiwifruit seedlings under drought stress (**Figure 4**), which was inconsistent with previous studies (Liang et al., 2019; Xia et al., 2020).

Under drought stress, plants increase water use efficiency mainly by closing stomata to reduce transpiration, but preventing carbon dioxide from entering mesophyll cells leads to a decrease in photosynthetic rate Pn, which is known as a stomatal limiting strategy (Du et al., 2018). Our results also confirmed a synchronously decline of Pn, Gs, Tr, and Ci under drought stress. However, the application of melatonin and AM did not significantly promote Pn, Gs, Tr, and Ci under drought stress, which was different from previous results (Liang et al., 2019), possibly because the weakened effect due to prolonged treatment time. Interestingly, the combined application of MT and AM had a greater effect on increasing Pn, Gs, Tr, and Ci, indicating an obvious superposition effect, as in study of tobacco (Liu et al., 2020).

Recent studies have found that melatonin enhances plant uptake of nutrients. For example, the concentrations of nitrogen, potassium, copper, iron, and zinc in grape fruits were improved by MT supplemental (Xia et al., 2021). Mycorrhiza can obviously promote the absorption of N, P and Fe, make the seedlings more conducive to nutrient growth and alleviate the damage caused by stress to plants (Chandrasekaran, 2022). In this study MT and AM both increased significantly the concentration of P in seedling leaves under watering and drought condition, importantly, which contributed to drought tolerance enhancement (Begum et al., 2020; Bechtaoui et al., 2021). PHT1 (phosphate transporter 1) family genes play an important role in regulating plant growth and coping with stress (Cao et al., 2020). Our results showed that the expression of all six PHT1 genes significantly increased after MT and AM induction. It was speculated that MT and AMF treatment can alleviate drought damage by inducing high expression of *PHT1*s to improve P uptake.

In addition to promoting plant uptake of nutrients in plants, melatonin supplementation also altered the composition of bacterial and fungal communities in the soil, alleviating the effect of apple diseases (Li et al., 2018). Herein, we further confirmed that melatonin could not only promote the uptake of soil nutrients by itself, but also expand plant uptake of distal soil nutrients by strengthening the symbiosis between arbuscular mycorrhizal fungi and roots and increasing colonization. However, different from the previous study on tobacco (Liu et al., 2020), melatonin and mycorrhizal seem to have no additive effect on nutrient absorption, especially in the mineral elements Fe, Zn and Cu, whose contents in the mixed treatment group decreased instead. It is speculated that they may have some competitive or antagonistic mechanism in their nutrient absorption to avoid metal poisoning.

In our study, melatonin and mycorrhiza have additive effect on improving plant growth under drought, and increasing antioxidant enzyme activity, chlorophyll content, osmotic regulating substance, photosynthetic capacity, and reducing MDA and REL. This is largely due to their different mechanisms for performing functions. As a broad-spectrum antioxidant, melatonin itself has the ability to remove ROS and prevent its accumulation in cells (Yu et al., 2018; Arnao and Hernández-Ruiz, 2019). At the same time, it can improve the activity of antioxidant enzymes and the content of antioxidant substances under adversity conditions, thus greatly reducing the damage caused by drought stress (Xia et al., 2020). On the other hand, melatonin, as an auxin precursor, has a similar function of promoting plant growth (Hernandez-Ruiz et al., 2005; Szafrańska et al., 2013). While mycorrhiza reduced the stress damage mainly by promoting nutrient absorption capacity, improving root function (Lenoir et al., 2016). Because extrapular-root mycelium formed a huge mycelium network in the soil, increasing root xylem conductance and improving the adaptability of water transport (Abdalla and Ahmed, 2021; Sun et al., 2022). Most important, in this study, melatonin was found to promote the establishment of a symbiotic relationship between arbuscular mycorrhizal fungi and roots.

## Conclusion

Under drought conditions, AM inoculation was more effective than MT in promoting root biomass, soluble sugar content, and absorption of N and Fe nutrients. MT has a better effect in improving shoot biomass and the activity of antioxidant enzymes to remove ROS accumulation. Moreover, combination of mycorrhizal and MT promotes the root systems to form a symbiotic relationship. In addition, the combination of AM and MT treatment had an additive effect on improving plant growth, antioxidant enzyme activity, chlorophyll content, osmotic regulating substance, photosynthetic capacity, and reducing MDA and REL. Our results indicated that MT and AM fungi have a synergistic effect on improving drought tolerance, which provides a new strategy for the widespread application of biological modulators to improve crop drought tolerance.

## Data availability statement

The original contributions presented in the study are included in the article/supplementary material, further inquiries can be directed to the corresponding author/s.

## Author contributions

HX and DL conceived the idea and designed the research; CY, YL, ZH and YG performed the experiments with the help of YL, HD and XT; CY, JW and XL analyzed the data; CY and HX drafted the manuscript; YuL and DL revised the manuscript, other authors provided suggestions for the revision of the manuscript. All authors contributed to the article and approved the submitted version.

## Acknowledgments

This work was supported by fund received from the Sichuan Science and Technology Program (2022YFH0049 and 2022-YF05-00655-SN). We also thank the fund support from the Engineering Technology Research Center of Biological Resources Conservation and Utilization of Inner Mongolia (21400-222526).

## Conflict of interest

The authors declare that the research was conducted in the absence of any commercial or financial relationships that could be construed as a potential conflict of interest.

## Publisher’s note

All claims expressed in this article are solely those of the authors and do not necessarily represent those of their affiliated organizations, or those of the publisher, the editors and the reviewers. Any product that may be evaluated in this article, or claim that may be made by its manufacturer, is not guaranteed or endorsed by the publisher.
