# Supplementary material for: Melatonin and arbuscular mycorrhizal fungi synergistically improve drought toleration in kiwifruit seedlings by increasing mycorrhizal colonization and nutrient uptake
Source: Front Plant Sci. 2022 Dec 1;13:1073917. doi: 10.3389/fpls.2022.1073917 (PMC9752077; doi:10.3389/fpls.2022.1073917)

Melatonin and arbuscular mycorrhizal synergies improved drought resistance in kiwifruit seedlings

Hui Xia ^#^, Chunguo Yang ^#^, Yuqi Guo, Jie Wei, Yunxuan Lang, Yan Liang, Xinbo Tian, Honghong Deng, Lijin Lin, Jin Wang, Xiulan Lv, Dong Liang*

College of Horticulture, Sichuan Agricultural University, Chengdu 611130, China

^#^ These authors contributed equally to this work

^*^ Corresponding author

Dr. Dong Liang

*Email: [liangeast@sicau.edu.cn](mailto:liangeast@sicau.edu.cn.com)

Tel.: +86-28-86291136

College of Horticulture,

Sichuan Agricultural University,

Huimin Road 211^#^,

Chengdu 611130,

China

**Table S1.** DNA sequences of PCR primers and their melting temperature (T_m_) used in quantitative real-time PCR (qRT-PCR) for determination of antioxidant enzyme gene copy number in *Actinidia chinensis* under different treatments.

| Gene_id | Gene name | Primer sequence (5’-3’) | T_m_ (^o^C) |
| --- | --- | --- | --- |
| Achn054861 | *SOD* | F: AAAGGCGGGCTAGGGTTAGG  R: TGGAAGATCCGGGAGCGATA | 59.04  58.51 |
| Achn052701 | *SOD[Cu-Zn]* | F: GCGGGTGACCTGGGAAACAT  R: AGGCTCTGCCGACGACTGAA | 57.87  61.82 |
| Achn387421 | *POD12* | F: CTGCCCAGCACTAGACACAA  R: GTCCTGGTCGGACGTAAAAA | 58.03  54.53 |
| Achn111691 | *POD42* | F: CCGAACGCGGTCCAGTATGT  R: TTGGTCCTCTTGTCGGTGGC | 59.55  58.29 |
| Achn310611 | *CAT1* | F: ACCTGAGTGCCCTTTAAGCC  R: TTTGGGTATGAACGAGTTGG | 57.02  52.94 |
| Achn029621 | *CAT6* | F: CTACATCCGCATCACCTTCG  R: TATCAGATTCGCTCCCGTCA | 57.66  57.28 |
| EF063572 | *Actin* | F: TGCATGAGCGATCAAGTTTCA  R: TGTCCCATGTCTGGTTGATGA | 59.85  58.01 |

**Table S2.** DNA sequences of PCR primers and their melting temperature (T_m_) used in quantitative real-time PCR (qRT-PCR) for determination of phosphate transporter gene copy number in *Actinidia chinensis* under different treatments.

| Gene_id | Gene name | Primer sequence (5’-3’) | T_m_ (^o^C) |
| --- | --- | --- | --- |
| Achn359671 |  | F: TTTCGGTCCAAACGCAACC  R: CCCACAATCGCCCCTAACTT | 55.29  56.79 |
| Achn352381 |  | F: AGCCGCCCTCACTTACTACT  R: GCATCAGCCTTCGCCTTGT | 58.49  59.08 |
| Achn334531 |  | F: CACGGAATTTCAGCAGCAT  R: GGATACCCTTTGTCGGTCTTA | 55.38  54.13 |
| Achn334521 |  | F: TCTACCGCTTTTGACCACG  R: CATCTTCATCCGCCAATAGTA | 55.83  54.72 |
| Achn193781 |  | F: CAGCCGACCGACAAGACAA  R: AAACAACATCCCCACGAAAT | 57.87  52.09 |
| Achn046121 |  | F: ATGAGCCCGTTCTTTCCAC  R: GGCATTTTCATTCGCCAGTA | 54.09  54.83 |

**Table S3.** Effects of drought stress, AM fungus inoculation and exogenous melatonin application on AM colonization (MC), spore density (SPD) and hyphal length density (HLD) in roots or rhizosphere soils of *Actinidia chinensis*.

| Treatments | | AM fungal status | | | | | |
| --- | --- | --- | --- | --- | --- | --- | --- |
|  |  | MC (%) | | SPD (number/g) | | HLD (m/g) | |
| WW | -MT | 65.7±0.76b | | 6.27±0.27c | | 2.34±0.08b | |
|  | +MT | 75.3±2.33a | | 7.58±0.09b | | 3.77±0.17a | |
| DR | -MT | 52.9±1.66c | | 6.50±0.13c | | 1.62±0.03c | |
|  | +MT | 72.7±2.99a | | 9.14±0.16a | | 2.28±0.04b | |
| Significance | | *F* | *P* | *F* | *P* | *F* | *P* |
| DR | | 45.754 | **<0.001** | 115.524 | **<0.001** | 283.708 | **<0.001** |
| MT | | 0.037 | 0.853 | 191.064 | **<0.001** | 93.469 | **<0.001** |
| DR × MT | | 68.612 | **<0.001** | 77.754 | **<0.001** | 378.828 | **<0.001** |

The values (means ± SD, *n* = 5) followed by the same letter in the same column do not differ significantly at *P* < 0.05 according to Duncan's multiple range test. MC, AM colonization; SPD, spore density; HLD, hyphal length density; WW, well-watering; DR, drought stress; -MT, non-melatonin application; +MT, melatonin application. Boldface indicates significant effects of DR, MT and DR × MT on MC, SPD and HLD by two-way ANOVA at *P* < 0.05.


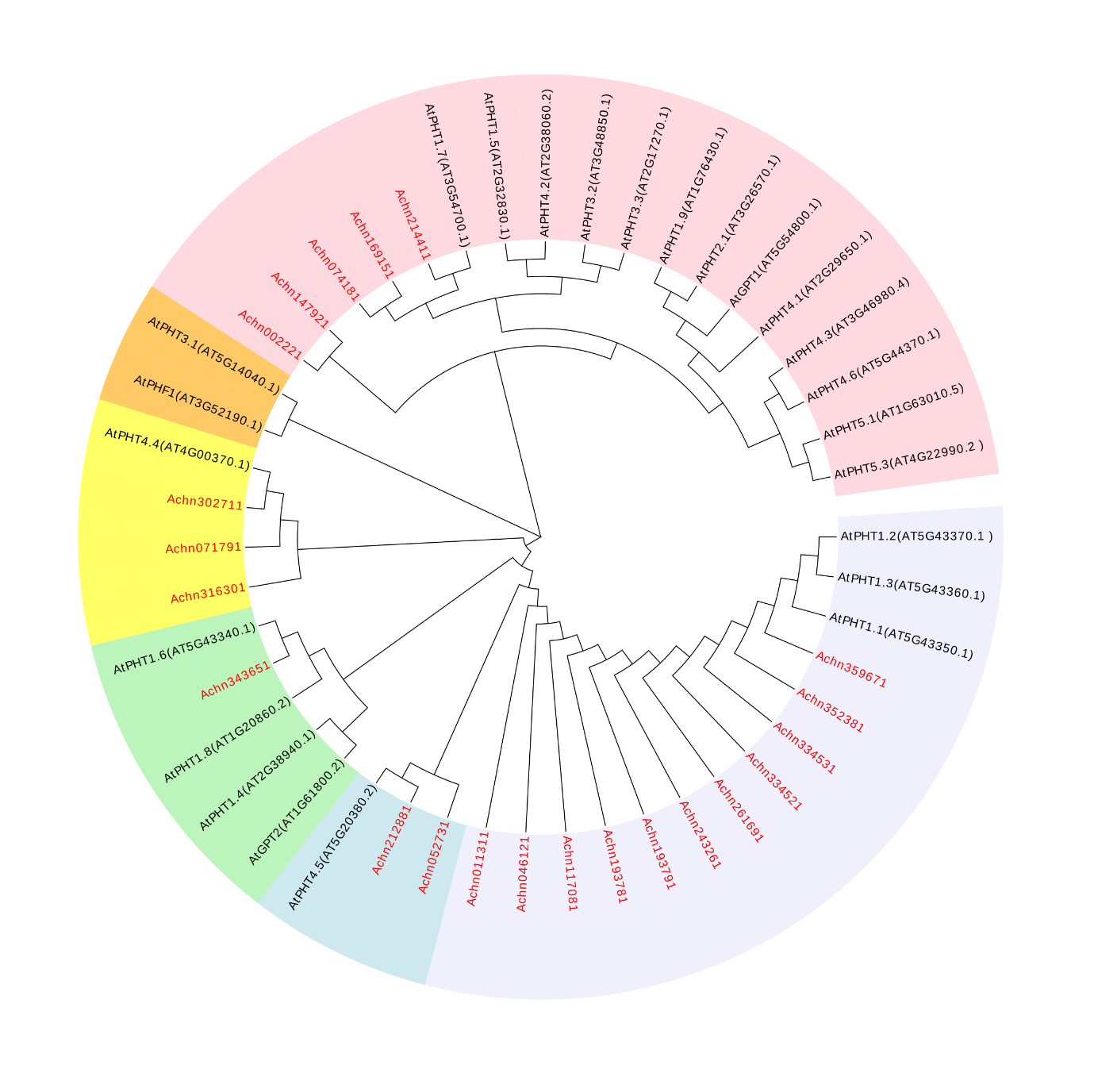


**Figure. S1 Unrooted phylogenetic tree of *Actinidia chinensis* and *Arabidopsis thaliana* PHT1 homologs proteins.**

Notes:

***Arabidopsis thaliana*:**

AtPHT1;1(AT5G43350.1)、AtPHT1;2(AT5G43370.1)、AtPHT1;3(AT5G43360.1)、AtPHT1;4(AT2G38940.1)、AtPHT1;5(AT2G32830.1)、AtPHT1;6(AT5G43340.1)、AtPHT1;7(AT3G54700.1)、AtPHT1;8(AT1G20860.2)、AtPHT1;9(AT1G76430.1)、

AtPHT2;1(AT3G26570.1)、AtPHT3;1(AT5G14040.1)、AtPHT3;2(AT3G48850.1) 、AtPHT3;3(AT2G17270.1)、AtPHT4;1(AT2G29650.1)、AtPHT4;2(AT2G38060.2)、AtPHT4;3(AT3G46980.4)、AtPHT4;4(AT4G00370.1)、AtPHT4;5(AT5G20380.2)、AtPHT4;6(AT5G44370.1)、AtPHT5;1(AT1G63010.5)、AtPHT5;3(AT4G22990.2 )、

AtGPT1(AT5G54800.1)、AtGPT2(AT1G61800.2)、AtPHF1(AT3G52190.1)；

***Actinidia chinensis*:**

Achn002221、Achn011311、Achn046121、Achn052731、Achn071791、Achn074181、Achn117081、Achn147921、Achn169151、Achn193781、Achn193791、Achn212881、Achn214411、Achn243261、Achn261691、Achn302711、Achn316301、Achn334521、Achn334531、Achn343651、Achn352381、Achn359671.


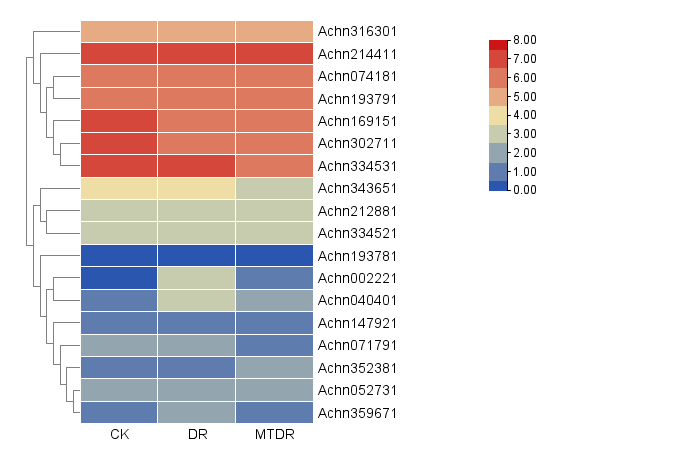


**Figure.S3 Gene expression heat map of PHT1 family expression in *Actinidia chinensis* with melatonin applied under drought stress.**

**Figure. S3** Pathway proposed for understanding the beneficial effects of exogenous melatonin on drought stress tolerance in *Actinidia chinensis*. The negative effects of drought stress on plant growth mainly involve a combination of ion injury, osmotic damage and reactive oxygen species (ROS) accumulation (brown arrows). Arbuscular mycorhrizal (AM) fungus inoculation can help the host plants to cope with the detrimental effects of drought stress via increasing photosynthetic rate, improving antioxidant enzyme activities and decreasing H_2_O_2_ and MDA contents (blue arrows). Moreover, exogenous melatonin application is able to further enhance the beneficial effects of AM fungi on host plant tolerance through promoting AM fungal growth, protecting photosynthesis, increasing expression of antioxidant enzymes, and thereby resulting in enhanced plant growth (yellow arrows).


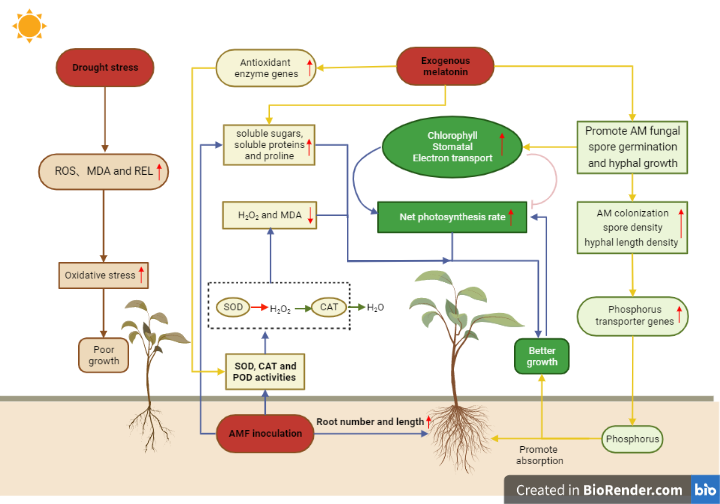

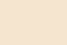

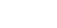

Supplement: Supplementary file 1 [file DataSheet_1.docx]
